# Adverse Childhood Experiences and Anxiety Symptoms in Adolescents during COVID-19

**DOI:** 10.1192/j.eurpsy.2023.483

**Published:** 2023-07-19

**Authors:** A. Marini, I. Farmakopoulou, M. Theodoratou, E. Gintoni, K. Chalkiopoulos

**Affiliations:** ^1^Child and Adolescent Psychiatry, Medical School, Athens; ^2^Department of Educational and Social Sciences, University of Patras, Patras, Greece; ^3^Health Sciences, Neapolis University of Pafos, Pafos, Cyprus; ^4^Social Sciences, Hellenic Open University, Patras; ^5^Psychology, University of Ioannina, Ioannina; ^6^Department of Science Management and Technology, University of Patras, Patras, Greece

## Abstract

**Introduction:**

Adverse Childhood Experiences (ACEs) are potential traumatic events that occur from birth to the end of adolescence (0-18 years), including various types of neglect, abuse and violence in a child’s domestic and community life. Experiencing Adverse Childhood Experiences (ACEs) is associated with the onset of anxiety in adolescence. According to recent studies, pandemic COVID-19 is a novel ACE that has been found to increase anxiety in adolescents.

**Objectives:**

To investigate the relationship between ACEs and COVID-19 in the development of anxiety in adolescence

**Methods:**

Α cross-sectional study was designed in a community sample of 248 adolescent boys and girls, aged 12 to 15 years (Mean: 13.5 years), from five High Schools in Eastern Attica. Four Questionnaires were used: 1) Demographic Questionnaire, 2) State-Trait-Anxiety-Inventory for Children - STAIC, 3) Adverse Childhood Experiences Questionnaire, and 4) Impact of COVID-19 Questionnaire.

**Results:**

The results demonstrated a strong correlation between the total number of ACEs and Anxiety (Trait and State) in adolescence (Trait Anxiety: rho=,37, p <0.001, State Anxiety: rho=,29, p<0.001).Girls scored significantly higher in Trait (U=4353, p <0.001) and State Anxiety (U=5822.5, p = 0.014), presenting higher anxiety compared to boys. Finally, a significant relationship was observed between the number of ACEs and the impact of COVID-19 (β=0.025, p <0.001).

**Conclusions:**

The findings of the present study can be used to design and implement future effective, preventive and therapeutic programs for adolescents with anxiety symptoms, who have experienced the multitude of Adverse Childhood Experiences and the COVID-19 pandemic during their adolescence.

**Disclosure of Interest:**

None Declared

